# Proliferation does not contribute to murine models of renin cell recruitment

**DOI:** 10.1111/apha.13532

**Published:** 2020-07-18

**Authors:** Omar Guessoum, Momna Zainab, Maria Luisa S. Sequeira‐Lopez, R. Ariel Gomez

**Affiliations:** ^1^ Department of Biology University of Virginia Charlottesville VA USA; ^2^ Department of Pediatrics University of Virginia Charlottesville VA USA; ^3^ Child Health Research Center University of Virginia Charlottesville VA USA

**Keywords:** cell cycle, homeostatic threat, JG Cells, transcriptome

## Abstract

**Aim:**

Renin cells are essential for regulation of blood pressure and fluid‐electrolyte homeostasis. During homeostatic threat, the number of renin cells in the kidney increases, a process termed as recruitment. It has been proposed that recruitment occurs by proliferation, yet no systematic studies have been performed. We sought to determine the extent to which proliferation contributes to the recruitment process.

**Methods:**

Mice were subjected to recruitment before analysing the renin cells’ cell cycle. For acute threats, we subjected SV129 and C57Bl6 mice to a low sodium diet plus captopril. Tissue sections from treated mice were co‐stained for proliferation markers (Ki67, PCNA, pH3 and BrdU) and renin. Chronic recruitment was studied in deletion models of aldosterone synthase and angiotensinogen through co‐immunostaining and counting mitotic figures in periodic acid‐Schiff‐stained sections. Finally, RNA‐seq of renin cells isolated from recruited mice was performed to study mitotic signature.

**Results:**

Mice subjected to low salt and captopril displayed increases in renin cell number (312 ± 40 in controls to 692 ± 85 in recruited animals, *P*<.0001), 10‐fold increases in renin mRNA and fourfold increases in circulating renin. Co‐staining these kidney sections for proliferation markers revealed negligible proliferation of renin cells (<2%), indistinguishable from control animals. Similarly, chronic models of recruitment—aldosterone synthase KO and angiotensinogen KO—had negligible proliferation. Additionally, the transcriptome of recruited renin cells revealed overall downregulation of mitotic pathways when compared to proliferative cell lines.

**Conclusion:**

Acute and chronic physiological threats to homeostasis produced a distinct increase in renin‐synthesizing cells, but we found no evidence to suggest the involvement of proliferation.

## 1 INTRODUCTION

Regulation of blood pressure and fluid‐electrolyte homeostasis is key to the survival of animals with a closed circulatory system.^1^ The Renin‐Angiotensin‐System (RAS) is responsible for such regulation and the tightly regulated production and secretion of renin from juxtaglomerular cells (JG) of the kidney constitute the system's rate‐limiting step. Renin catalyses the formation of angiotensin I from angiotensinogen in a cascade leading to angiotensin II generation which elicits vasoconstriction and sodium reabsorption ultimately resulting in increased blood pressure. A fascinating phenomenon involving JG cells is that, under conditions in which homeostasis is threatened and blood pressure falls, the number of renin cells in the kidney increases.^2^, ^3^ This process has been termed ‘recruitment’ to indicate that cells distant from the glomerulus, along the afferent arterioles, and sometimes in the glomerular mesangium and peritubular interstitium express renin.^4^ However, the extent to which hyperplasia of renin cells contributes to the increase in renin cell number as opposed to transformation of existing cells to the renin phenotype is not fully understood.

**FIGURE 1:**
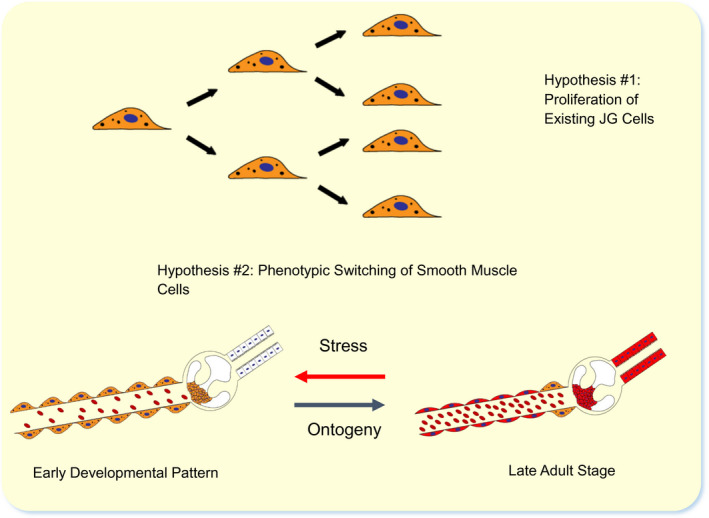
Proliferation vs plasticity. Hypotheses put forward to explain the increase in renin cell number seen during stress. One possibility is proliferation and migration of existing renin cells to bring about an overall increase in renin cell number. An alternative hypothesis is phenotypic switching of neighbouring cells in the kidney. A systematic evaluation of proliferation in the recruited kidney has not been performed to date

The initial description of the increased number of renin cells along the arterioles in response to decreased perfusion and hypotension found no evidence of DNA synthesis as assessed by tritiated thymidine uptake.^5^ This led to the conclusion that the increased number of renin‐synthesizing cells was caused by ‘metaplasia’ of existing vascular cells rather than cell proliferation. This finding is supported by our previous work indicating that cells descended from a renin‐expressing progenitor are capable of re‐expressing renin.^4^ In addition, it has been reported that recruitment involves cells exhibiting de novo renin expression as determined by dual colour lineage tracing in a process termed neogenesis.^6^ However, and in contrast to these findings, other groups proposed that proliferation of JG cells plays a key role during recruitment and use the term hyperplasia to describe the process^7^, ^8^, ^9^, ^10^ (Figure 1). Despite these conflicting findings, a systematic assessment of renin cell proliferation with various proliferation markers and transcriptomic analysis has, to date, not been conducted to conclusively resolve this question.

**FIGURE 2:**
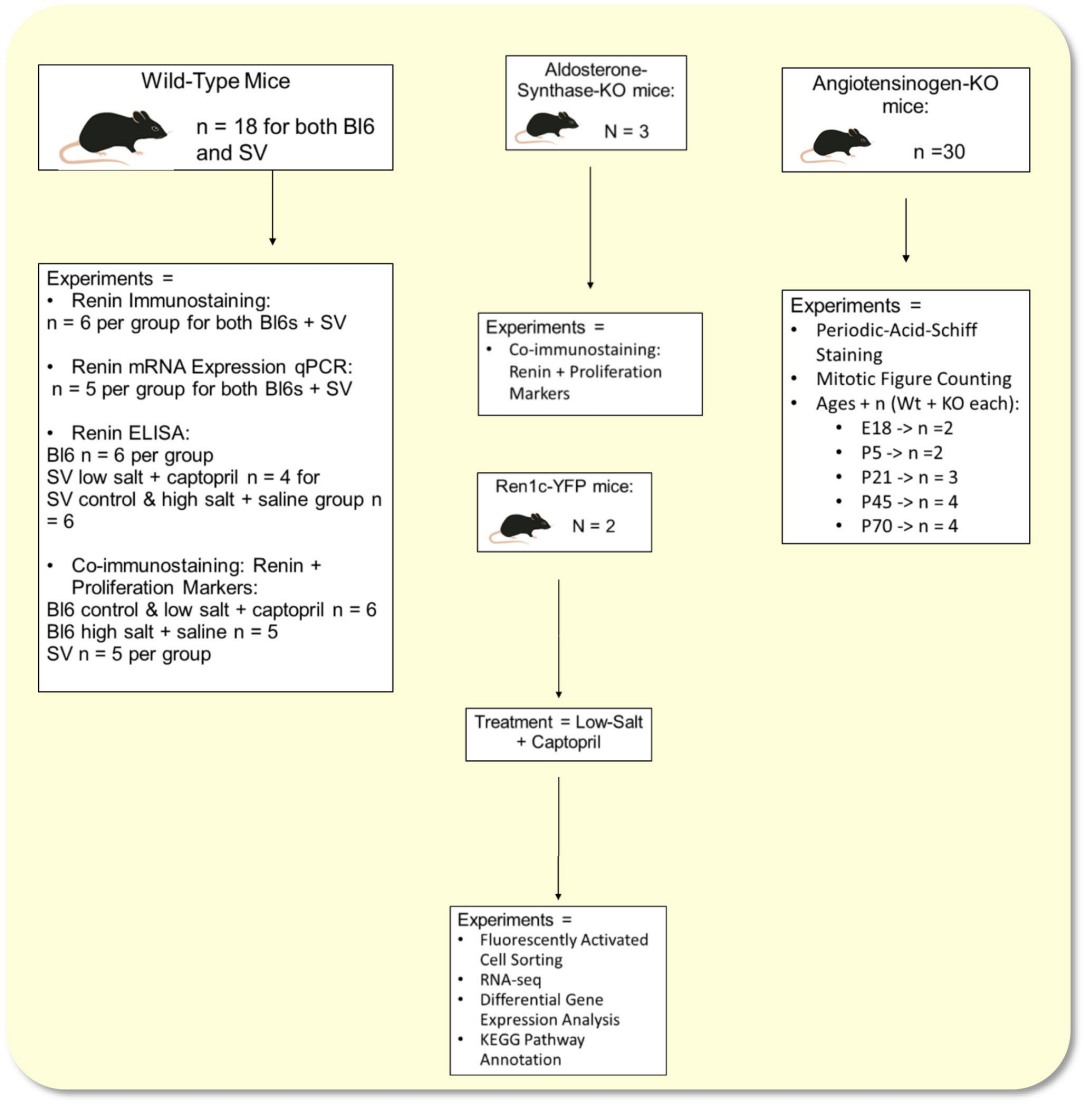
CONSORT diagram of mouse subjects. Consort diagram illustrating the genotype and number of mice utilized for each experiment throughout this paper

The prevailing hypothesis in the study of renin recruitment involves phenotypic switching/transdifferentiation of existing vascular cells into renin‐expressing cells (Figure 1). In this study, we use several models in which blood pressure and fluid homeostasis are threatened to stimulate the increase in renin cell number to test the hypothesis that proliferation plays a role in the recruitment process (Figures 1 and 2). We performed co‐immunostaining for renin and various proliferation markers to determine the number of renin cells undergoing cell division during basal physiological conditions and under homeostatic threats. Additionally, we utilized Fluorescence‐Activated Cell Sorting (FACS) to purify recruited renin cells. These cells were subjected to RNA‐seq to examine their transcriptome and compare them with proliferative cell lines to determine if they had a mitotic signature. Taken together, these assays allowed us to measure the contribution of proliferation to renin cell recruitment.

## 2 RESULTS

**FIGURE 3:**
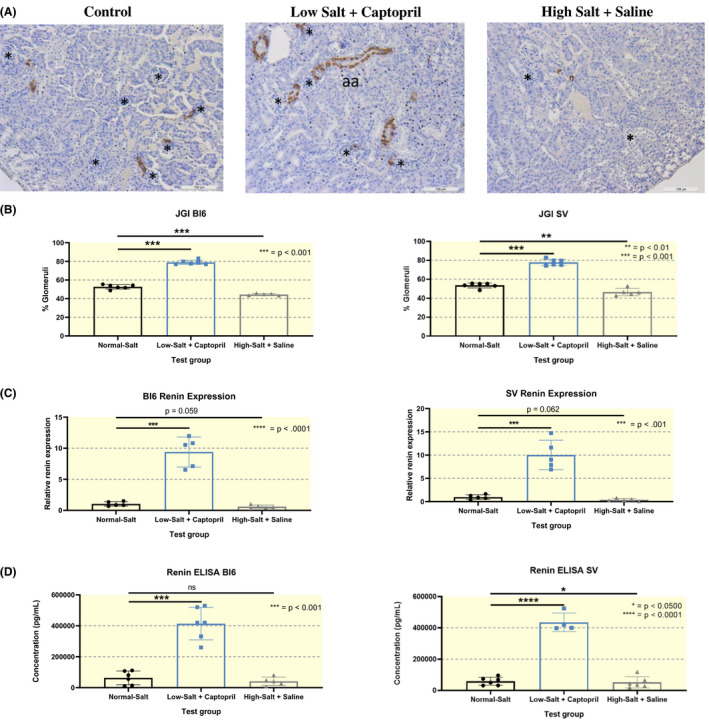
Changes in renin expression constitute a physiological response against homeostatic threats. A, Kidney sections of mice stained for renin protein, marker of renin cells, show a notable increase in renin cell number in the captopril‐treated group. aa, afferent arteriole. *Glomeruli. B, Juxtaglomerular Index (JGI) quantification for each condition is consistent with staining results. JGI, number of JGAs positive for renin staining/total number of glomeruli × 100. C, Relative renin expression normalized to GAPDH using kidney cortices from study animals. D, Changes in renin mRNA expression are reflected in circulating/active renin protein levels in the circulation to respond to loss of homeostasis. n ≥ 4 and all points on graphs represent individual mouse test subjects

To determine the extent to which proliferation occurs during acute homeostatic threats, we treated mice with a diet low in sodium and captopril in their drinking water for a week to stimulate the recruitment of renin‐expressing cells. We performed these experiments on both the C57Bl6 and SV129 strains, two strains historically used for studying renin cells.^11^, ^12^ Furthermore, we also treated mice with a diet high in salt and saline in the drinking water. Staining for renin protein confirmed the effects of the treatments as there was an increase in vascular, renin‐expressing cells in the low salt + captopril condition, whereas there was a clear reduction in renin‐expressing cells in the high salt condition (Figure 3A). Expansion of renin cell number largely occurred in afferent arterioles (aa), whereas in the control group, renin cells remained localized to the juxtaglomerular area. Conversely, a diet high in sodium results in diminished renin expression and fewer renin‐expressing cells. This was also confirmed by the juxtaglomerular index (JGI) for each of these conditions (JG Index: controls = 52.7%, low salt = 78% and high salt = 44%, *P* < .01) (Figure 3B). Furthermore, qPCR for renin mRNA on kidney cortices from each of these conditions paralleled the staining results as there was an increase of 10‐fold in renin mRNA in the low salt plus captopril group (*P* < .0001) and a tendency towards reduction with a threefold decrease in the high salt diet group (*P* = .059) (Figure 3C). Finally, to confirm that increased expression of renin constituted a functional, physiological response, we measured circulating renin levels by ELISA and found a highly significant increase in circulating renin in mice subjected to the low salt diet plus captopril (*P* < .001) which confirmed the above findings (Figure 3D). Circulating renin measurements for mice treated with high salt and saline revealed a significant increase in the SV129 strain but not for Bl6 animals although the trend tended towards reduction in renin levels (from 63641 to 41943 pg/mL, *P* = .36). Finally, we collected urine from the treated mice and quantified the sodium content which was reduced from ~250 to 20 mmol/L in mice subjected to low sodium + captopril and significantly elevated to up to 400 mmol/L in the high salt + saline treatment (Figure S1, *P* < .01). These results are indicative of the efficacy of the treatments as mice treated with low salt and captopril attempt to retain sodium to expand extracellular fluid volume and raise blood pressure while mice exposed to high salt attempt to excrete it to lower blood pressure. Taken together, these results indicate that homeostasis was perturbed, resulting in recruitment of renin cells in mice treated with low salt and captopril and repression of renin in mice treated with high salt and saline.

**FIGURE 4:**
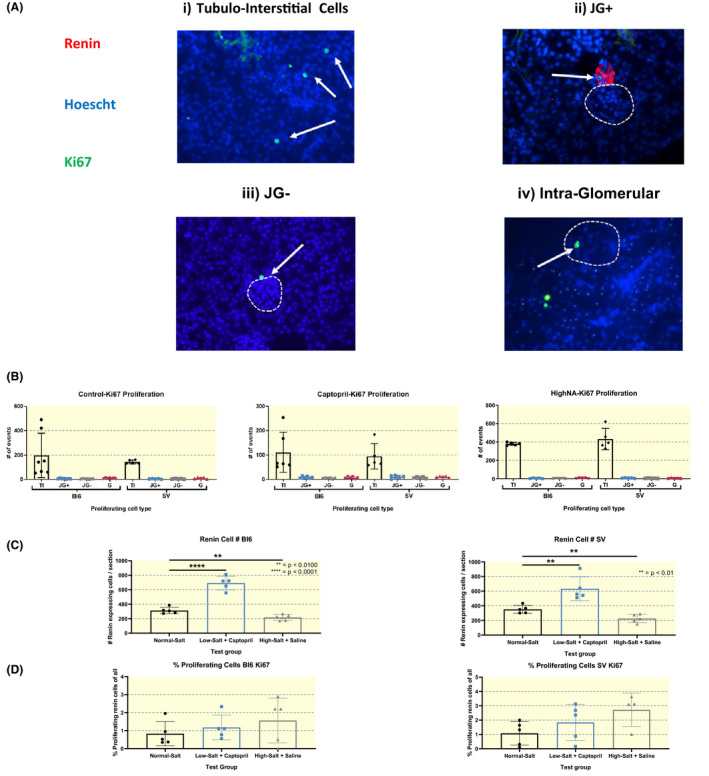
Quantification of proliferating renin cells reveals proliferation is infrequent and not significantly increased during acute homeostatic threats. A, Kidney sections of test groups co‐stained for renin protein and Ki67. Proliferating cells were identified in: (i) Tubular cells, (ii) JG cells positive for renin, (iii) JG cells negative for renin and (iv) Intra‐glomerular cells. Dotted lines indicate glomeruli. B, Quantification of all proliferating events in kidney sections organized by cell type across control, recruited and high salt + saline conditions. C, Quantification of total number of renin cells per section in each condition. D, Normalization of proliferating Ki67 + renin‐positive cells obtained by dividing the number of dually positive (Ki67+, renin+) cells by the total number of renin + cells per section × 100. n ≥ 5 and all points on graphs represent individual mouse test subjects

Having demonstrated that the mice responded to the treatments appropriately, we proceeded to perform co‐immunofluorescence for renin and Ki67 to identify dividing renin cells in each condition (Figure 4). All proliferation events in the kidney were counted and assigned to one of several groups: tubulointerstitial (Figure 4Ai), JG cells positive for renin expression (Figure 4Aii), JG cells negative for renin expression (Figure 4Aiii) and glomerular cells (Figure 4Aiv). Figure 4B illustrates the results of counting all the events from two non‐consecutive kidney sections for each treatment and reveals that >90% of the proliferation occurs in the tubular compartment while other cell types (including renin‐expressing cells) rarely undergo cell division. Because the different conditions result in vastly different numbers of renin‐expressing cells, we sought to normalize the number of proliferating renin‐positive cells. This was accomplished by dividing the proliferating renin cell number by the total number of renin cells for mice in each treatment. Control mice with more than 300 renin cells were found to have six dividing renin cells on average while recruited mice had fewer than 10 dividing renin cells out of 692 (Figure 4C and D). Therefore, the proportion of dividing renin cells under basal conditions was low (<2%) and was not significantly increased, that is remained unchanged during recruitment (*P* = .44). Considering that recruitment generates an increase from 312 to 692 renin cells between the control and low salt + captopril condition, this degree of proliferation cannot account for the increased number of renin cells observed. In addition, we performed co‐staining for renin and other proliferation markers, such as pH3 and PCNA, and observed identical results, further emphasizing the lack of proliferation during stress (Figure S2). Therefore, these findings suggest that proliferation plays a negligible role in the increase in renin cell number observed after a whole week of a sustained homeostatic threat.

**FIGURE 5:**
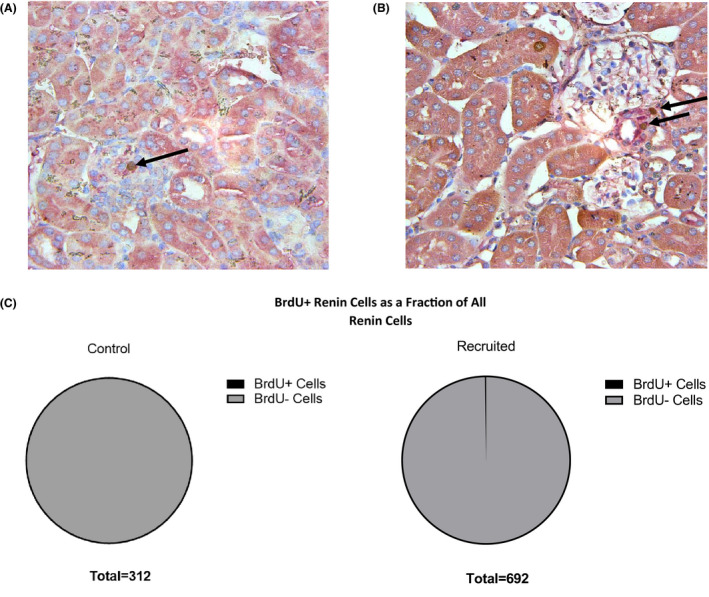
Co‐staining Renin and BrdU Reveals Negligible Proliferation of Renin Cells During Recruitment: (A) A proliferating recruited mesangial cell expressing renin. B, Proliferating renin cells in the juxtaglomerular area. C, Quantification of all renin‐expressing cells displayed in a pie chart with BrdU ± cells illustrated. n = 3 representing mouse subjects

To identify renin cells undergoing DNA synthesis and actively entering S‐phase, we injected BrdU into the control and recruitment groups and performed double immunostaining for renin and the BrdU analog. Figure 5A and B depict examples of proliferating renin cells identified in the mesangium and JGA respectively. All such events were counted and divided by the total number of renin cells per section and the results are reported in Figure 5C. Although the presence of BrdU + renin cells was detected in the recruited samples, which was notably absent in the controls, the prevalence of such events was exceedingly rare and constituted less than 0.2% of all renin cells. Therefore, the DNA synthesis assay strongly suggests that proliferation does not contribute significantly to the increase in renin cell number during acute recruitment.

**FIGURE 6:**
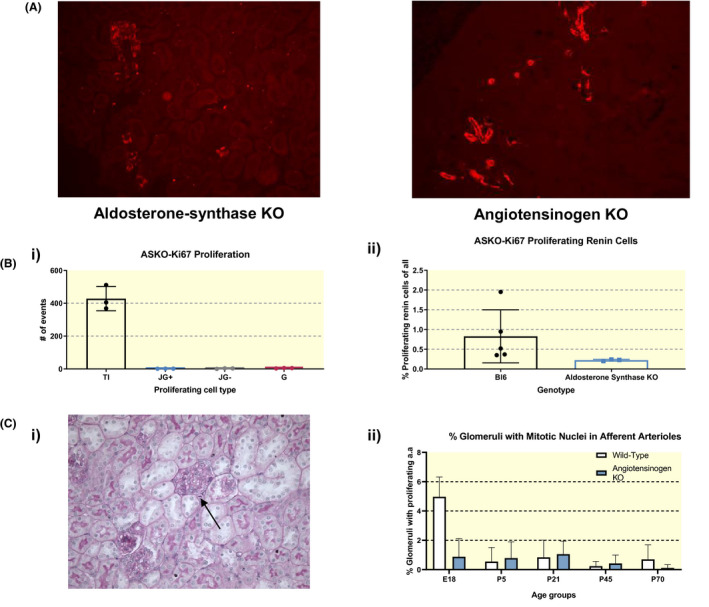
Proliferation of renin cells under chronic conditions: (A) Immunofluorescence for renin protein in two conditions of chronic stress: aldosterone synthase KO and angiotensinogen KO. B, (i) Quantification of all proliferating events in aldosterone synthase KO tissues co‐stained for renin and Ki67 as in Figure 3. (ii) Normalization of proliferating renin cells by total renin cells by: # of JG + cells/Renin Cell # per section × 100. C, (i) PAS staining in angiotensinogen KO tissues to identify mitotic figures in vessels as shown by the arrow. (ii) Quantification of mitotic figures across several post‐natal ages and compared to wild‐type Bl6 controls. n ≥ 2 and points on graphs represent individual mouse test subjects

Having established that renin cells do not proliferate to restore homeostasis in response to a sustained physiological threat of an entire week, we sought to determine whether proliferation plays a role in more prolonged/chronic models of renin recruitment. Specifically, we measured proliferation in mice homozygous for aldosterone synthase deletion and mice homozygous for angiotensinogen deletion.^13^, ^14^, ^15^ Each of these models exhibits increased renin expression and heightened renin cell number as a result of chronic homeostatic imbalance (Figure 6A). We performed co‐immunostaining for the proliferation markers described above and renin to quantify proliferating renin cells in aldosterone synthase KO tissues (Figure 6Bi), and found comparable results with very low/negligible levels of proliferation (less than 1%) in this model (Figure 6Bii). In addition, we performed PAS staining in angiotensinogen KO tissues across different ages throughout development to visualize nuclei undergoing mitosis (Figure 6Ci). These were quantified and normalized to glomerular number before comparison to wild‐type Bl6 controls which revealed no significant increases in proliferation. Therefore, in models of subacute and chronic homeostatic threats accompanied by massive increases in the number of renin cells, we found no evidence of increased proliferation. We must conclude, therefore, that renin cells do not undergo proliferation to restore homeostasis.

**FIGURE 7:**
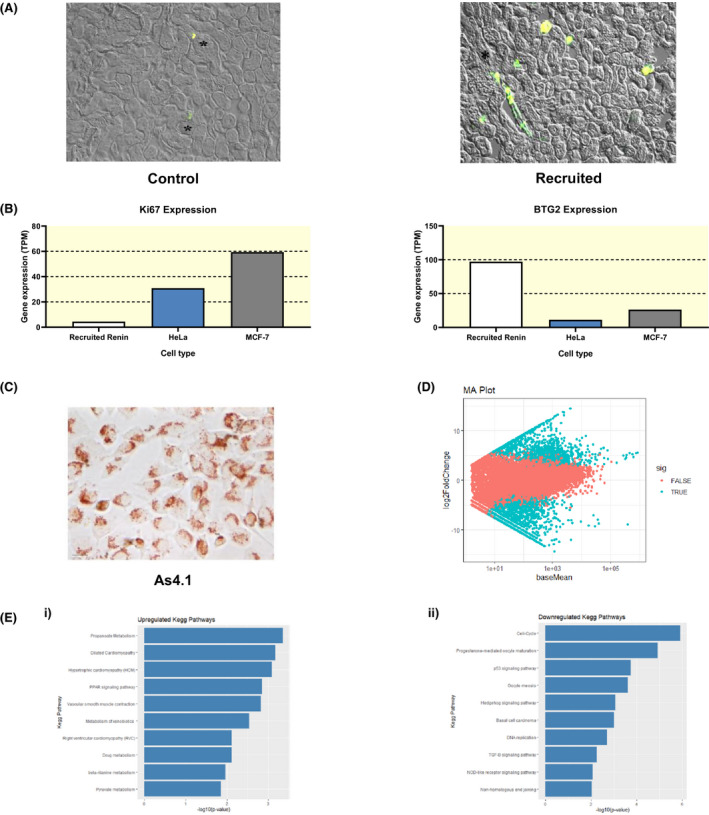
Transcriptome analysis of isolated cells; expression of proliferation‐associated genes does not increase during physiological threats: (A) Frozen tissue sections of mice bearing a transgenic YFP which labels renin cells and reports the activity of the renin promoter. Conditions observed were basal physiological conditions and after subjection to homeostatic threats. A clear expansion of the number of YFP + renin cells is seen under stress B, Transcriptome profiling of renin compared to cell lines known to have a high degree of proliferation. (i) Expression of Ki67 proliferation marker. (ii) Expression of BTG2/Anti‐Proliferation Factor 2. C, Tumoural cell line As4.1s, which constitutively express renin, stained with neutral red. Adapted from reference 16. D, Using the R package DeSeq2, 2830 genes were identified as differentially expressed between these two cell types. The manta‐ray (MA) plot depicts the expression level and significance of the genes used. E, (i) Depiction of up/downregulated pathways in recruited renin cells when compared to the renin‐expressing, cancerous As4.1 cell line. (ii) The most downregulated pathway in this comparison is the cell cycle

Finally, we analysed the transcriptome of recruited renin cells to determine whether they expressed genes associated with progression through the cell cycle. We challenged C57Bl6 mice bearing a *Ren1C‐YFP* transgene which labels all renin‐expressing cells with YFP using the conditions described above to stimulate recruitment^16^ (Figure 7A). YFP+/renin‐expressing cells were then isolated by FACS and processed for RNA‐seq to measure the expression of cell cycle‐associated genes such as Ki67. Expression of Ki67 in both recruited and untreated YFP + cells was exceedingly low (<5 transcripts per million). Furthermore, data on cell types known to have a high proliferative capacity were extracted from the ENCODE database and used to compare to the expression levels in our cells (Figure 7B). Expression of Ki67 was about fivefold higher in the HeLa and MCF‐7 breast cancer tumoural lines than in the recruited renin cells. Additionally, BTG2, a known tumour suppressor,^17^, ^18^ was highly enriched in the recruited cells but sharply diminished in the proliferative cell lines indicating the anti‐mitotic state of these cells.

Our initial analyses focused on cell cycle genes such as tumour suppressors but, to expand our study and increase its value, we decided to perform a whole transcriptome evaluation to determine differentially regulated pathways. For this analysis, we used RNA‐seq data from As4.1 cells,^16^ a tumoural cell line which expresses renin and may serve as a better basis for comparison when looking at changes occurring at the level of the transcriptome (Figure 7C). We used the DeSeq2 package to find differentially regulated genes between the recruited renin cells and the As4.1 cells, which were then examined to determine up/downregulated pathways using the DAVID‐KEGG Annotation. About 2830 genes were found to be differentially regulated (Figure 7D), but the most downregulated pathway in the recruited renin cells relative to the As4.1s was the cell cycle, confirming our previous data (Figure 7Eii). Upregulated pathways, by comparison, include pathways known to play an important role in renin cells such as the PPAR signalling pathway, metabolism of xenobiotics involving genes such as AKR1B7 etc (Figure 7Ei). Therefore, our results mirror both our findings regarding proliferation as well as previous literature involving renin cells. These results improve upon and support our initial findings of proliferation playing an inconsequential role in the increase in renin cell number seen during recruitment.

## 3 DISCUSSION

The question of renin cell proliferation in response to homeostatic threat has been subject to contradictory findings in the field of renin research. This work finds very little to no contribution of proliferation to the increase in renin cell number seen during stress. To our knowledge, ours is the first work to systematically assess proliferation using various assays including various proliferation markers co‐stained with renin, DNA synthesis assays, mitotic figure observation and transcriptomic profiling. We used different strains of mice alongside both acute and chronic models of homeostatic threat and failed to detect a significant degree of proliferation in any of these models. Because none of these methods or models support proliferation having anything beyond a minute effect on the increase in renin cell number, we conclude that the increase in renin cell number is likely due to phenotypic switching of neighbouring, vascular smooth muscle, mesangial and interstitial cells to adopt a renin‐expressing cell identity. Furthermore, we also treated mice with a diet high in salt and saline in the drinking water, conditions known to stimulate an increase in proliferation throughout the kidney as the bioenergetic burden on tubules is increased.^19^ As expected, we observed a large increase in the proliferation of the tubular compartment and interstitial cells, yet renin cell proliferation remained low in response to a broad range of physiological challenges indicating that other mechanisms may be responsible for the increase in renin cells in response to stress. Considering that a single mouse kidney section contains between 50,000 and 100,000 cells, the exceedingly low proportion of renin cells undergoing proliferation serves to highlight that mechanisms other than proliferation govern the recruitment process.^20^ In addition, the high expression of BTG2, the downregulation of cell cycle pathways and the exceedingly rare occurrence of reninomas suggest that the mitotic programme of these cells is highly repressed. The reasons for this repression are unclear but one may speculate that a cell critical for maintaining homeostasis and survival would be under constant and precise regulation to prevent their tumourigenesis. Although the historical difficulties in isolating renin cells (low cell number, etc) have limited studies of the cell cycle and its components in the renin cell, emerging technologies and techniques may help overcome such limitations.

During early embryonic life, renin expression is widespread throughout the kidney and in the vasculature in particular where it is thought to aid in the formation of vessels. However, as ontogeny proceeds, expression of renin gradually recedes until it is spatially restricted to the juxtaglomerular area. However, when blood pressure falls or during dehydration, renin is expressed by cells in the vasculature in a pattern mimicking the foetal pattern.^21^ It can be assumed that numerous cells re‐developing renin expression is an evolutionarily advantageous trait as it allows a rapid response to loss of homeostasis. Considering that the RAS plays a crucial role in survival by ensuring constant perfusion and oxygen delivery to critical organs, the ability to rapidly address loss of homeostasis would dramatically increase the chances of survival. This line of thought is supported by the fact that the RAS and the ability of renin cells to be recruited are conserved across various species ranging from fish to mammals.^22^ Increasing the number of cells engaging in the production of renin exponentially increases the quantity of renin released allowing acute physiological threats to be dealt with in a timely manner ensuring survival.

From an evolutionary standpoint, one may speculate that proliferation of renin cells, on the other hand, is a costly and ineffective strategy to cope with homeostatic threats. After threats have been addressed, renin expression must quickly fall to avoid adverse cardiovascular events and/or due to excessively high blood pressure. If proliferation and migration of JG cells truly occurred, those cells would have to undergo rapid apoptosis and be phagocytosed by nearby cells to maintain the structural integrity of the kidney. These events have not been observed. From a bio‐energetic standpoint, this process is unlikely as it requires large amounts of energy and resources to synthesize the new cells to be ultimately destroyed. Furthermore, the temporal requirements for proliferation are inappropriate to serve as a mechanism for addressing acute homeostatic threats. The shortest time required for mammalian cells to undergo cell division is estimated to be between 24 and 48 hours.^23^ Acute threats, such as dehydration, can be lethal within a much shorter time frame making proliferation an ineffective mechanism for coping with such threats. These arguments, in addition to all the supporting evidence demonstrating a lack of proliferation, strongly refute proliferation as a mechanism to restore homeostasis neither for acute or chronic threats.

Having established that proliferation is a minimal contributor to the recruitment process, future studies should focus on elucidating the molecular mechanisms governing the ability of cells in the vasculature to adopt the renin cell identity. Our group has previously demonstrated, using ATAC and ChIP‐seq, that native JG cells as well as recruited renin cells have activating marks at various loci which regulate the expression of core genes of the renin cell identity.^16^ Additionally, we have also found a set of transcription factors that are expressed in recruited renin cells and it is likely that these are involved in changing the epigenetic landscape and activating the necessary genes for recruitment. It remains to be determined what is the native state of the recruited cells before recruitment and while they are still smooth muscle, mesangial and pericyte in nature. Whether they are prepared to adopt the renin cell identity by having poised epigenetic marks at particular loci or if they must undergo a complete remodelling of their epigenetic landscape remains unknown but represents an exciting avenue for future research.

In summary, we used multiple methods in an attempt to detect proliferation of renin cells during homeostatic threats and found that the cells do not undergo significant proliferation to produce the dramatic expansion of renin cells seen during stress. Thus, based on available experimental data, we must conclude that phenotypic switching of renin lineage cells into renin cells is the main contributor to elevating renin levels to cope with physiological threats.

## 4 MATERIALS AND METHODS

### 4.1 Animals

To study proliferation of renin cells, we studied mice with manipulations known to affect renin expression. Mice aged 2‐3 months were studied and included wild‐type C57BL6 mice (Jackson Laboratories) and several genetically modified mouse strains, including mice with deletion of aldosterone synthase (AS), which display a prominent increase in the number of renin cells along the arterioles,^24^ mice with deletion of the angiotensinogen gene, which show a dramatic expansion of renin cells in vessels and pericytes,^13^, ^25^ and mice with expression of YFP driven by the Ren1c promoter (*Ren1c‐YFP* mice), which reports activity of the renin promoter.^26^ A minimum of five animals per group were examined with a mixture of males and females. Physiological challenge to induce increased renin expression was performed using a 7‐day treatment with low sodium diet (0.1%, 7034, ENVIGO) plus captopril added to the drinking water (0.5 g/L) (20). We also challenged a group of mice with a diet high in sodium (3.2% Na, TD.92012) and saline drinking water. Mice were anaesthetized intra‐peritoneally with tribromoethanol (300 mg/kg) prior to harvest and terminated by cervical dislocation subsequent to tissue harvesting. All animals were handled in accordance with the National Institutes of Health guidelines for the care and use of experimental animals, and the study was approved by the Institutional Animal Care and Use Committee of the University of Virginia.

### 4.2 Blood collection

After anaesthetizing, a 23‐gauge needle was inserted into the cardiac stalk and used to draw blood which was collected into BD microtainer tubes containing EDTA (BD 365974‐1). Blood was then centrifuged at 1000 g for 10 minutes to separate blood components.

### 4.3 Urine collection

Mice were scruffed and gentle, manual pressure was applied to the transabdominal area to elicit urination. The urine was collected into 1 mL Eppendorf tubes and submitted to testing for sodium content.

### 4.4 Immunohistochemistry for renin

Subsequent to treatment, kidneys were harvested and fixed in Bouin's solution and prepared for immunostaining, as previously described.^27^ Kidney sections were deparaffinized, hydrated and blocked in 3% BSA and normal goat serum before exposure to our antibody against renin [rabbit polyclonal 1:500 dilution].^4^ Visualization was performed using the appropriate Vectastain Elite ABC kit (Vector Laboratories), and sections were counterstained with haematoxylin. Kidney sections were mounted and examined under a microscope (Leica DFC 480) and imaged with a digital camera (Leica DFC310 FX).

### 4.5 Periodic acid‐Schiff's staining

Bouin's fixed kidney sections were deparaffinized and hydrated before immersion in 0.5% periodic acid solution (Sigma # P‐7875) for 10 minutes. Sections were washed three times with ddH2O following oxidation and subsequently immersed in Schiff's reagent (Sigma # S‐5133) for 20 minutes. Sections were then washed to remove excess Schiff's reagent and nuclei were counterstained with haematoxylin for 2 minutes followed by running tap water. Sections were then incubated in lithium carbonate to enhance staining contrast for 5 seconds followed by washing. Finally, sections were dehydrated and mounted before imaging.

### 4.6 DNA synthesis studies

To detect actively dividing cells which were replicating their DNA, 5‐Bromo‐2‐deoxyuridine (BrdU) was dissolved in PBS at 10 mg/mL and injected into mice intra‐peritoneally. To detect both rapidly dividing cells and their slower counterparts, injections were performed twice; 2 hours prior to harvest for the former and 24 hours prior to harvest for the latter.

### 4.7 Double Immunostaining for Renin and BrdU

Kidneys and small intestines (used as a positive control to confirm BrdU incorporation) were fixed in formalin, embedded and sectioned. Staining was performed using diaminobenzidine for BrdU and VIP‐purple for renin as described previously in^27^ and using the anti‐renin [rabbit polyclonal 1:500 dilution]^4^ and the rat anti‐BrdU antibody (Abcam ab6326).

### 4.8 Morphometric measurements

The juxtaglomerular apparatus (JGA) index was calculated as the number of renin‐positive JGA/total number of glomeruli and expressed as a percentage. To determine the number of renin‐expressing cells per section, we counted the number of renin‐positive cells in each JGA plus the number of renin‐positive cells along the arterioles with visible glomeruli attached to them. To determine the number of proliferating cells in angiotensinogen KO tissues, we stained samples from ages E18 (n = 2), P5 (n = 2), P21 (n = 3), P45 (n = 4) and P70 (n = 4) with the periodic acid‐Schiff stain and counted proliferation events in afferent arterioles leading to glomeruli. We then divided this value by the total number of glomeruli and expressed the result as a percentage.

### 4.9 Immunofluorescence for proliferation markers

To detect proliferating renin cells in formalin‐fixed tissue sections, we used co‐immunofluorescence with an anti‐rat renin antibody 1:200^28^ alongside proliferation marker antibodies against pH3 (Cell Signalling; #9701, 1:200), Ki67 (Novus NBP‐54791, 1:200) or PCNA (Cell Signalling; 13110S, 1:1000). Sections were deparaffinized and rehydrated using xylene and a succession of alcohols. Antigen retrieval was conducted by boiling for 10 minutes in 10 mmol/L sodium citrate and pH 6.0. Kidney sections were incubated overnight at 4°C with primary antibodies. Sections were then exposed to Alexa Fluor‐488 donkey anti‐rabbit and Alexa Fluor‐568 donkey anti‐goat (1:500 dilution) secondary antibodies and counterstained with Hoechst (Invitrogen). Kidney sections were mounted and examined under a microscope (Leica DFC 480) and imaged with a digital camera (Leica DFC360 FX). An average of two kidney sections separated by 70‐100 µmol/L was counted for proliferative events within the entire section for each biological replicate. Additionally, the number of co‐localizations of renin and proliferation markers was counted and used to compute the % of proliferating renin cells for each condition.

### 4.10 Plasma renin

Plasma renin concentration was determined using ELISA following the manufacturer's instructions (RayBiotech) as we previously described.^29^

### 4.11 Quantitative PCR

To measure mRNA for renin and GAPDH in RNA from isolated kidney cortices, we conducted quantitative PCR (qPCR), as described previously (20), by reverse transcribing mRNA isolated from cells and amplified using the following primers: GAPDH [5– AAC TTT GGCATT GTG GAA GGG CTC‐3=(forward) and 5– ACC AGT GGATGC AGG GAT GAT GTT–3=(reverse)], and Renin [5– ACA GTA TCC CAA CAG GAG AGA ACA AG −3=(forward) and 5– GCA CCC AGG ACC CAG ACA −3=(reverse)].

### 4.12 Isolation of renin cells

*Ren1c‐YFP* mice at 2 months of age, alongside physiological untreated controls, were treated with a low sodium diet (0.1%, 7034, ENVIGO) plus captopril added to the drinking water (0.5 g/L) for 7 days. At the end of the treatment period, YFP + cells were isolated by FACS and cells were used to perform RNA‐seq.

### 4.13 RNA‐seq

For renin cells isolated from *Ren1c‐YFP* mice, the Clontech SMARTer‐Seq Lysis, RT and PCR solutions (Mountain View, CA; Catalog No. 634833) were used to generate bulk cDNA samples (~400 cells from the resuspended cells post‐FACS). The cDNA was diluted with Fluidigm dilution buffer the following day and stored at −20°C. The samples were quantified using a Qubit 3.0 Fluorometer (Thermo Fisher Scientific). Samples underwent next‐generation sequencing library preparation as described in the Fluidigm C1 manual using an Illumina Nextera XT library prep and index kits (Catalog Nos. FC‐131‐1096 and FC‐131‐1002). Briefly, cDNA samples were diluted, tagmented and indexed with unique barcodes for downstream analyses. After the addition of indices and amplification, samples were multiplexed and cleaned using AMPure beads. All samples were sequenced on a HiSeq2500/4000 platform.

We used FastQC to assess the quality of FASTQ file reads. Prior to alignment, we removed low quality reads and adapter sequences using Trimmomatic 0.36. We aligned FASTQ reads to the GRCm38/ENSEMBL mouse genome using Salmon 0.7.2 and transcript‐level estimates of expression were scaled up to gene‐level estimates using the *Tximport* 1.2.0 R package, with the ‘lengthScaledTPM’ argument for abundance estimation.

### 4.14 KEGG pathway analysis

The DeSeq2 package was used to identify differentially regulated genes between As4.1 cells and recruited renin cells. Those genes were subjected to Kyoto Encyclopedia of Genes and Genomes (KEGG) pathway analysis to identify biologically relevant pathways. The Database for Annotation, Visualization and Integrated Discovery (DAVID) was used to integrate and annotate functional genomic information.

### 4.15 Statistical analysis

qPCR experiments were performed in triplicate, and qPCR measurements were conducted three times per replicate. All results are presented as means* ±* SD. Statistical significance between two groups was determined by unpaired Student's *t*‐test. A *P* ≤ .05 was considered significant.

## CONFLICT OF INTEREST

None declared.

## Supporting information

Fig S1

Fig S2

Fig S3

## Data Availability

The data that support the findings of this study are openly available in NCBI’s GEO database. The recruited renin cell RNA‐seq data discussed in this publication have been deposited in the NCBI GEO and are accessible through GEO Series accession number GSE146386. Human cell line data were obtained through GEO Series accession numbers GSE90237 and GSE86661. As4.1 cell line data were obtained through GEO Series accession numbers GSE117725.
