# Supplementary figures and images for: Proliferation does not contribute to murine models of renin cell recruitment
Source: Acta Physiol (Oxf). 2020 Jul 18;230(3):e13532. doi: 10.1111/apha.13532 (PMC7583373; doi:10.1111/apha.13532)

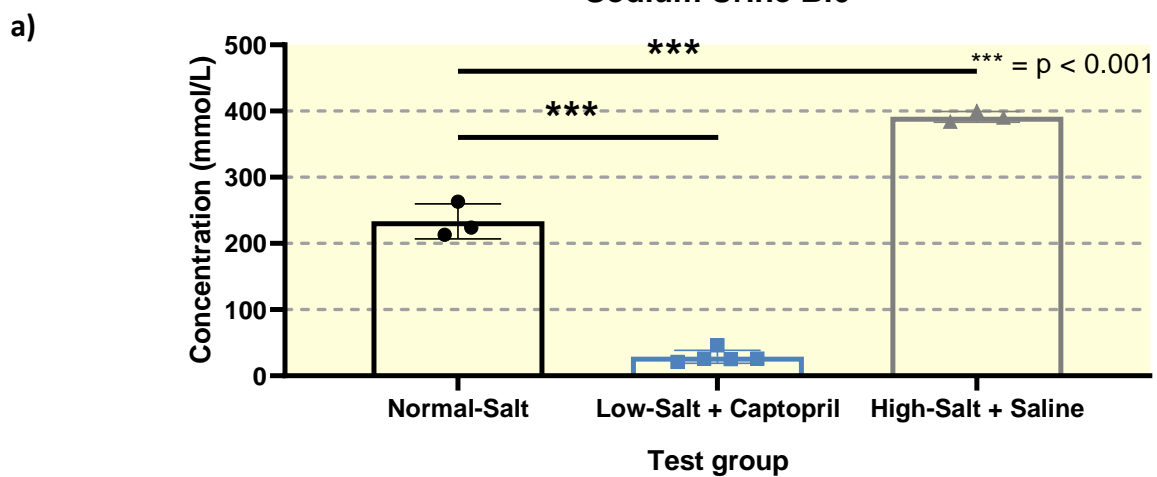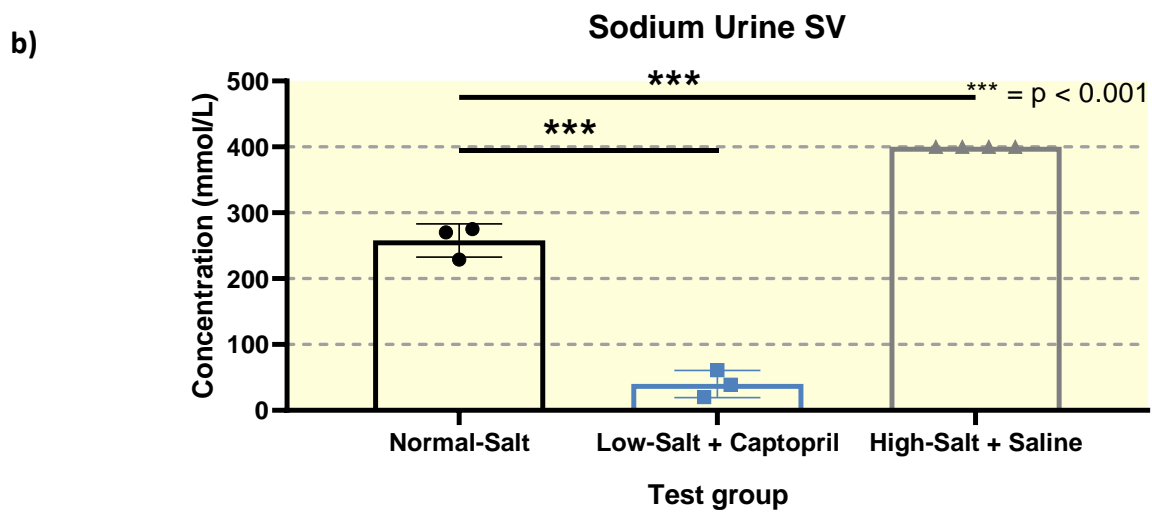

Supplement: Supplementary file 1 — Fig S1 [file APHA-230-e13532-s001.pdf]

a)

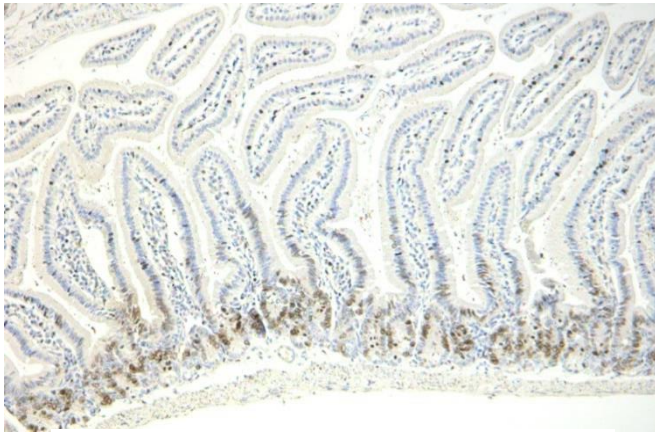

**BrdU**

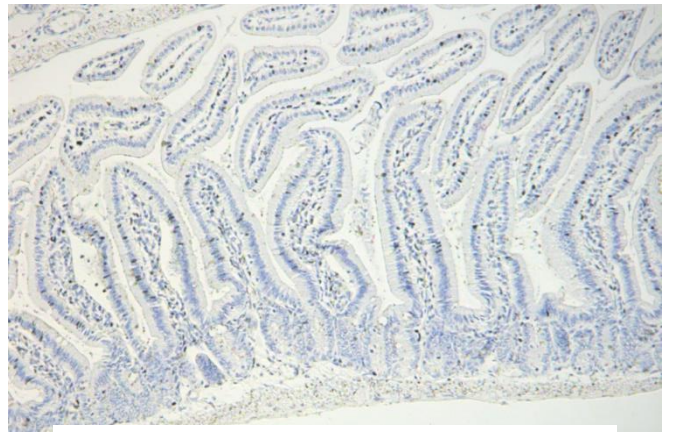

**Negative Control**

b)

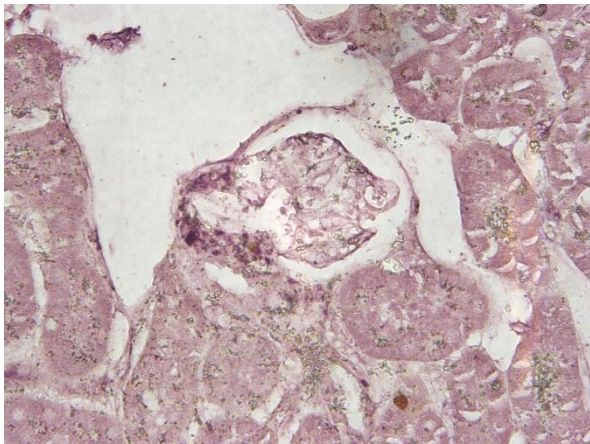

**BrdU + Renin Co-Immunostaining**

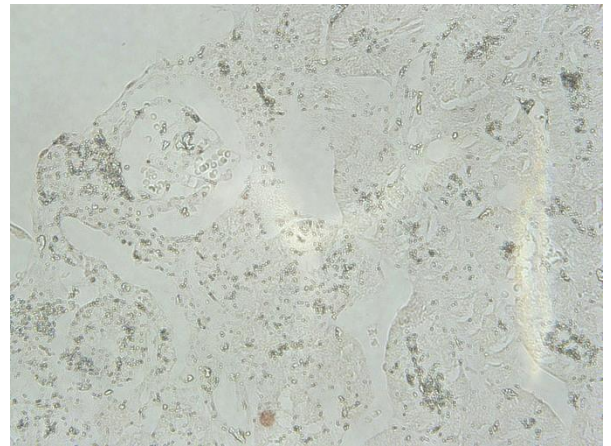

**BrdU Staining Alone**

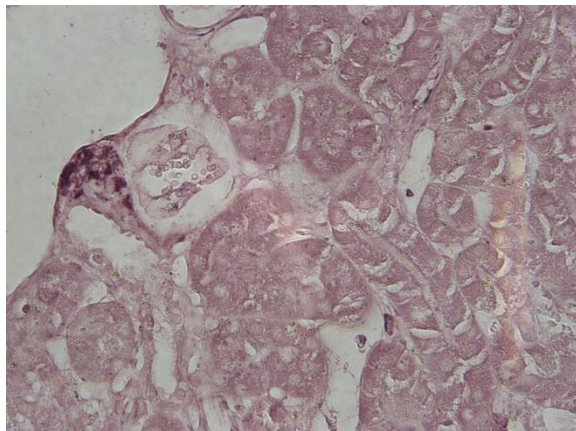

**Renin Staining**

Supplement: Supplementary file 3 — Fig S3 [file APHA-230-e13532-s003.pdf]
