# Supplementary material for: Proliferation does not contribute to murine models of renin cell recruitment
Source: Acta Physiol (Oxf). 2020 Jul 18;230(3):e13532. doi: 10.1111/apha.13532 (PMC7583373; doi:10.1111/apha.13532)
Supplement: Supplementary file 2 — Fig S2 [file APHA-230-e13532-s002.pdf]

a)

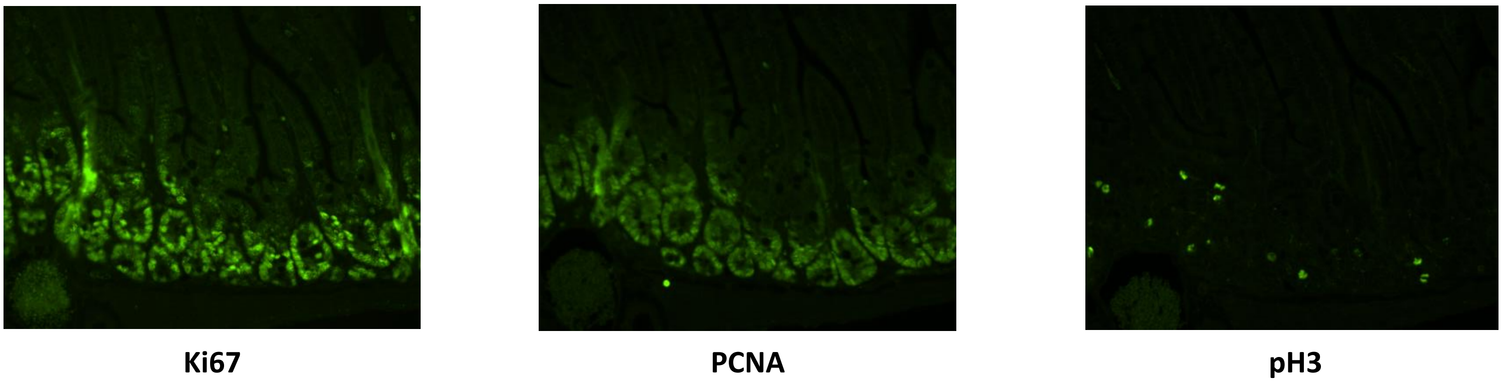
Ki67
PCNA
pH3

b)

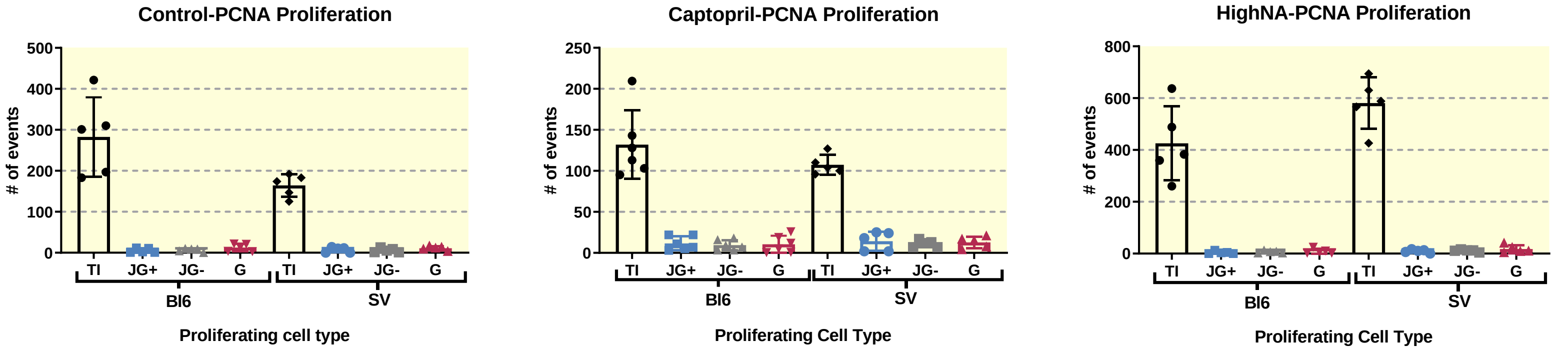
Control-PCNA Proliferation
# of events
500
400
300
200
100
0
TI
JG+
JG-
G
Bl6
SV
Proliferating cell type
Captopril-PCNA Proliferation
250
200
150
100
50
Proliferating Cell Type
HighNA-PCNA Proliferation
800
600

c)

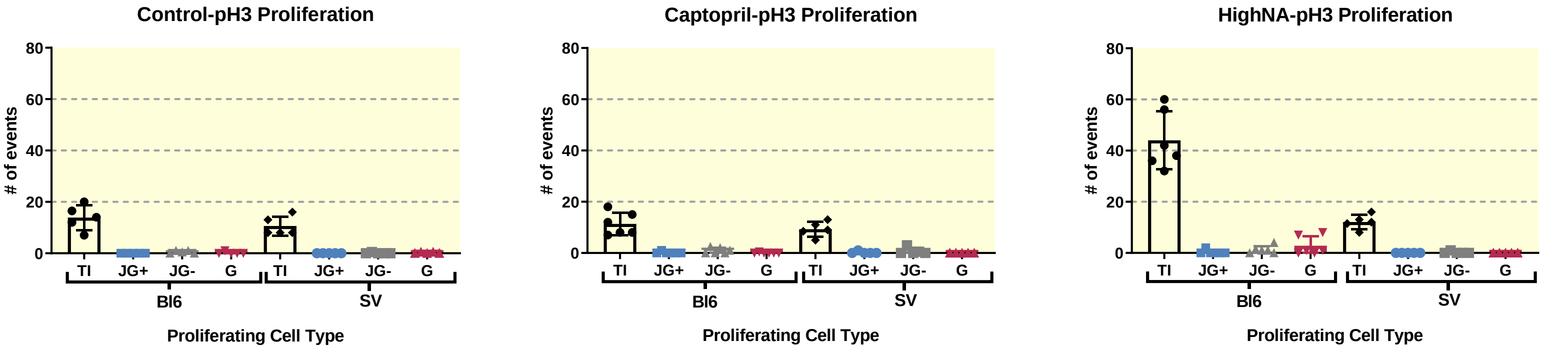
Control-pH3 Proliferation
# of events
80
60
40
20
0
TI
JG+
JG-
G
Bl6
SV
Proliferating Cell Type
Captopril-pH3 Proliferation
HighNA-pH3 Proliferation
